# *Orientia tsutsugamushi* in Lung of Patient with Acute Respiratory Distress Syndrome, France, 2013

**DOI:** 10.3201/eid2102.140860

**Published:** 2015-02

**Authors:** Emmanouil Angelakis, Gerome Patrick, Jean Michel Peloni, Pierre François Wey, Celine Perreal, Didier Raoult

**Affiliations:** Aix Marseille Université, Marseille, France (E. Angelakis, P.F. Wey, C. Perreal, D. Raoult);; L'Hôpital d’Instruction des Armées Desgenettes, Lyon, France (G. Patrick, J.M. Peloni, P.F. Wey)

**Keywords:** Orientia tsutsugamushi, bronchoalveolar lavage, acute respiratory distress syndrome, France, Laos, lung, scrub typhus

**To the Editor:** Pulmonary involvement is a well-documented complication of scrub typhus caused by *Orientia tsutsugamushi* (*1*). Lung involvement manifests as bronchitis and interstitial pneumonitis of various grades that progress to acute respiratory distress syndrome (ARDS), a serious complication that occurs in ≈11% of scrub typhus patients (*2*). The death rate among scrub typhus patients with ARDS can reach 25% (*3*). Older age, thrombocytopenia, and the presence of early pneumonitis have been proposed as risk factors for the development of ARDS in scrub typhus patients (*3*). We report the detection and culture of *O. tsutsugamushi* in a bronchoalveolar lavage specimen from a patient with scrub typhus–associated ARDS.

A 50-year-old woman from Lyon, France, was admitted to the hospital in November 2013 with fever (39°C), dizziness, diarrhea, dyspnea, and nonproductive cough. The woman, who had just returned from travel to a jungle in Laos, reported that the fever and diarrhea had begun immediately before her return home. Examination revealed that she had an oval eschar on her back and a faint maculopapular rash. Laboratory values showed elevated C-reactive protein and liver enzyme levels, lymphocytopenia, and thrombocytopenia. Extensive microbiological testing was done, including tests to rule out malaria, dengue, viral hepatitis, and leptospirosis; all results were negative. *Salmonella* sp. infection was suspected, and treatment with ofloxacin was started. 

On hospitalization day 5, the patient showed development of septic shock, renal failure, and ARDS. She was transferred to an intensive care unit, and treatment with ceftriaxone was started. On hospitalization day 6, a skin biopsy of the eschar (2 mm × 5 mm) and blood, serum, cerebrospinal fluid (0.5 mL), and bronchoalveolar lavage (1 mL) samples were obtained and sent to the National Reference Center for Rickettsiae (Marseille, France) for analysis. Total genomic DNA was extracted (Biorobot EZ1 Workstation; QIAGEN, Courtaboeuf, France) from 200 μL of each sample and used as template in a real-time PCR, which used primers and probes targeting a 47-kDa outer membrane protein gene, as described (*4*). Blood, skin biopsy, and bronchoalveolar lavage samples were positive for *O. tsutsugamushi*; the cerebrospinal fluid sample was negative. The serum sample was positive for *O. tsutsugamushi* by indirect immunofluorescence assay (serotypes Gilliam, Kuroki, Sennetsu, and Kawasaki) (*5*) and positive for *O. tsutsugamushi* IgM. Oral doxycycline (200 mg/day) was started on hospital day 7; the fever resolved 4 days later.

For culture, the positive samples were directly inoculated into monolayers of L929 cells, as described (*6*). Cultures of blood and skin biopsy samples were negative, but *O. tsutsugamushi* was isolated from the bronchoalveolar lavage sample after 40 days of culture (Figure); 500 μL of bronchoalveolar lavage fluid was used for culture. We performed PCR amplification and sequencing of the isolate, targeting a 372-bp fragment of the 56-kDa protein gene, and compared the sequences with *O. tsutsugamushi* 56-kDa protein–encoding gene sequences available in GenBank (*7*). The sequences showed 99% similarity with strains Jin/2012 and Zhou/2013 (GenBank accession nos. KJ001159 and KJ001163, respectively), which were obtained from febrile patients in Zhejiang Province, China, and have not been linked to a reference serotype (Technical Appendix Figure). In light of the test results and the patient’s recent travel to Laos, she was given a diagnosis of *O. tsutsugamushi* infection–associated ARDS.

**Figure:**
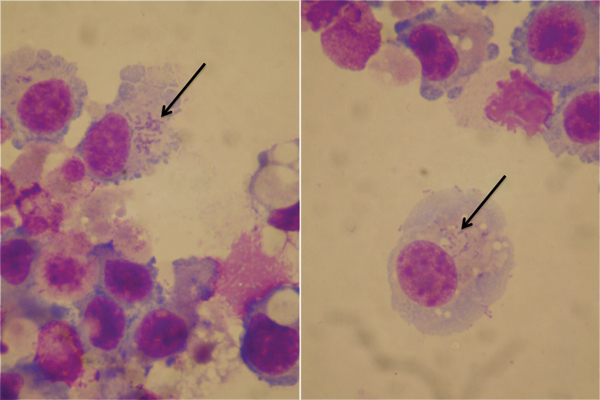
*Orientia tsutsugamushi* (arrows) in culture of bronchoalveolar lavage fluid from a patient with acute respiratory distress syndrome (Diff-Quick stain, VWR International, France). Original magnification ×100.

Our isolation of *O. tsutsugamushi* in bronchoalveolar lavage fluid from a patient with scrub typhus shows that this bacterium can be present in such samples. We also showed that skin biopsy and bronchoalveolar lavage samples can be used for the diagnosis of scrub typhus. To be suitable for culture, samples must be collected as early as possible in the disease course. In this case, blood and skin biopsy samples were obtained late in the disease, which may explain why *O. tsutsugamushi* was not isolated from these samples. Endothelial cells are the target cells of *O. tsutsugamushi* in the lung (*8*), and it has been proposed that ARDS in scrub typhus is associated with a cytokine increase as part of the immune response to *O. tsutsugamushi* infection (*9*). 

Rickettsial diseases are increasingly being diagnosed in international travelers: one report showed that 2% of imported fevers are caused by rickettsioses, and hospitalization was necessary for the 38% of *O. tsutsugamushi*–infected travelers (*10*). The diagnosis of rickettsial infections is challenging because many physicians are unfamiliar with these diseases. However, the diagnosis of scrub typhus in patients with ARDS is critical for initiating appropriate and timely doxycycline treatment. In the case reported here, a diagnosis of scrub typhus was not suspected even though the patient had compatible exposure and travel histories and clinical findings consistent with the disease. The delay in diagnosis led to a life-threatening condition for the patient. Physicians in areas where scrub typhus is nonendemic should have a high index of suspicion for rickettsial infections in patients with recent travel histories to areas where the disease is endemic and consider treatment with tetracyclines whenever rickettsial infection is suspected. Furthermore, the potential for aerosol transmission of *O. tsutsugamushi* from patients with scrub typhus–associated ARDS to health care workers should be evaluated.

Technical Appendix Phylogenetic tree for *Orientia tsutsugamushi* 56-kDa protein–encoding gene sequences obtained from GenBank.
