# Supplementary material for: Orientia tsutsugamushi in Lung of Patient with Acute Respiratory Distress Syndrome, France, 2013
Source: Emerg Infect Dis. 2015 Feb;21(2):373–5. doi: 10.3201/eid2102.140860 (PMC4313642; doi:10.3201/eid2102.140860)
Supplement: Technical Appendix — Phylogenetic tree for Orientia tsutsugamushi 56-kDa protein–encoding gene sequences obtained from GenBank. [file 14-0860-Techapp-s1.pdf]

Article DOI: http://dx.doi.org/10.3201/eid2102.140860

Orientia tsutsugamushi in Lung of Patient with Acute Respiratory Distress Syndrome, France, 2013

Technical Appendix

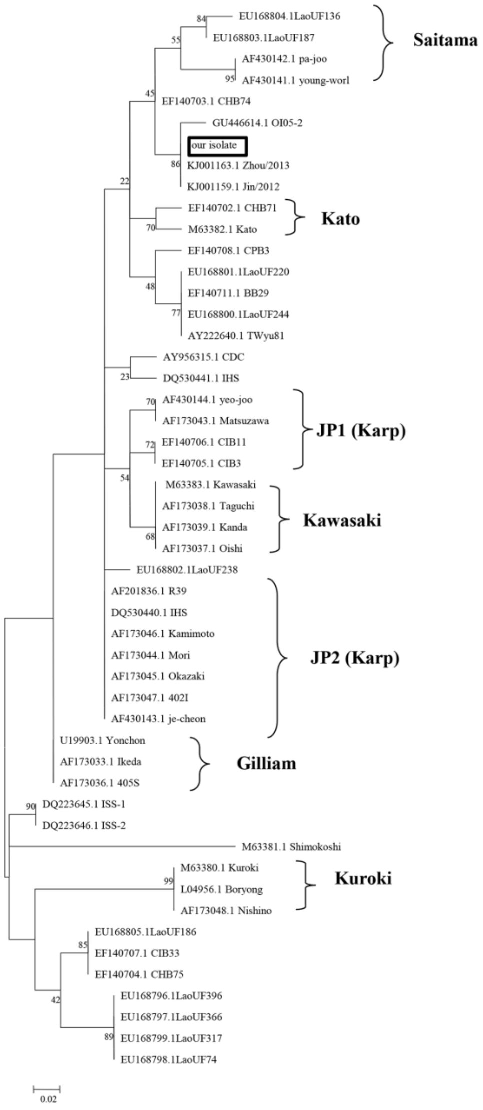

Online Technical Appendix Figure. Phylogenetic tree constructed by using the neighbor-joining method and MEGA software (http://megasoftware.net/) for *Orientia tsutsugamushi* 56-kDa protein-encoding gene sequences obtained from GenBank, as previously described (*1*). The

isolate from this study is indicated by a box labeled “our isolate.” Sequences are identified by GenBank accession number. Numbers at nodes represent bootstrap values based on 100 replicates. Scale bar represents 2% nucleotide sequence divergence. JP, Japan

Reference

1. Fournier PE, Siritantikorn S, Rolain JM, Suputtamongkol Y, Hoontrakul S, Charoenwat S, et al. Detection of new genotypes of *Orientia tsutsugamushi* infecting humans in Thailand. Clin Microbiol Infect. 2008;14:168–73. PubMed http://dx.doi.org/10.1111/j.1469-0691.2007.01889.x
